# Supplementary material for: Are carbon emissions trading and green financial instruments synergistic? -Comprehensive quantitative research based on content analysis
Source: PLoS One. 2024 Mar 7;19(3):e0298601. doi: 10.1371/journal.pone.0298601 (PMC10919601; doi:10.1371/journal.pone.0298601)
Supplement: S1 Data — (ZIP) [file pone.0298601.s001.zip › Data/地级市Z_F工作报告.zip!/╝¬┴╓/╝¬┴╓╩╨/└┤╫╘╣1⁄2╓┌║┼í╛┬φ┐╦╩2╛▌═°í┐_2014.docx]

各位代表：

　　现在，我代表市政府向大会报告工作，请予审议，并请政协委员提出意见。

　　一、2013年工作回顾

　　去年是新一届政府的开局之年，也是外部环境尤为复杂多变的一年。面对经济下行压力加大、筹融资紧张、财政收入增幅回落明显等诸多困难，我们在省委、省政府和市委的正确领导下，在市人大、市政协的大力支持下，团结带领全市人民，把稳增长作为核心任务，着力提高经济运行质量和效益，全面加大重点工作落实和服务力度，经济社会保持了平稳较快的发展态势。预计实现地区生产总值2630亿元，可比增长9%；规模工业增加值952亿元，增长9%；固定资产投资2125亿元，增长20%；招商引资到位资金950亿元，增长23%；社会消费品零售总额1062亿元，增长13.5%；地方级财政收入139.9亿元，增长17.6%；城镇居民人均可支配收入25598元，农民人均纯收入10413元，均增长16%。

　　（一）强化稳增长措施，总量在结构优化中实现扩充

　　实施“三动”战略成果显著。坚持投资拉动、项目带动和创新驱动战略，推动总量和结构的互促共进，三次产业结构调整到9.9：49：41.1；战略性新兴产业占规模工业比重提高2.8个百分点，与传统产业比达到28.7：71.3。以康乃尔化工硝酸、恒联技术改造为代表的一批传统产业改造升级项目建成投产，以锌镍二次电池、10万吨高性能改性塑料复合材料为代表的一批战略性新兴产业项目建设加快，以欧亚城市综合体、亚琦国际商贸城为代表的一批服务业项目顺利实施，以海王健康产业基地、中讯软件服务外包为代表的一批重点招商项目签约落地。全年在建3000万元以上项目1203项，其中亿元以上650项，重点招商项目350项。出台促进民营经济发展的一系列政策，大力组织实施“五大工程”，民营经济成为稳增长的重要支撑。全年新增“三上”企业205户，新生成民营企业3300户，民营经济主营业务收入、实缴税金分别增长20.7%和16.2%，民营经济贡献率首次突破50%。

　　工业经济保持平稳增长。以扭亏增盈为核心，全面加强产需衔接、银企保对接和要素保障，推动技改升级、新技术应用和建成项目达产达效，服务企业开拓市场、降低成本、强化管理，百户重点企业总体止跌企稳，十大行业保持不同幅度增长。全市规模工业实现产值3250亿元，利润55亿元，亏损企业减亏19亿元。

　　服务业发展步伐加快。4个省级服务业集聚区启动建设。地产品推介成效明显，电子商务服务平台初步建成，新兴商贸业态更趋丰富，城乡市场持续繁荣。深度开发文化旅游产品，建成19项重点文化旅游项目，以雾凇、冰雪、红叶、河灯、游江为主题的系列活动特色鲜明、影响扩大，旅游总收入增长25%。吉高物流园、晟驰汽车物流等一批物流重点项目建成使用。区域性云计算产业基地加快建设，中油数据中心建成，江南数据中心与7家金融机构实现业务合作。

　　现代农业稳步发展。积极推广高光效休耕轮作技术，粮食产量稳定在110亿斤以上。畜牧业和特产业产值分别增长8.9%和21.7%。实施方圆食品等145个重点农产品加工项目，新增市级以上龙头企业47户。品牌建设和推广力度加大，被命名为“中国粳稻贡米之乡”。农业综合生产能力增强，完成各类农田水利建设418项，农机综合作业率达到69%。大力培育新型经营主体，新发展家庭农场600个、农民专业合作社633个。土地流转面积占农村承包土地面积的14%。实施新农村建设项目326项，集体经济收入超百万元村达22个。永吉无规定疫病区运行良好。

　　（二）优化空间布局，区域综合发展能力明显提升

　　十大功能区集群效应凸显。坚持高标准规划，各功能区规划体系逐步完善。实施各类基础设施工程60项，完成投资60亿元，新建功能区空间框架快速拓展，成熟功能区承载能力进一步增强。强化对外开放，中新食品区中新合资开发管理公司组建完成，与泰国正大成功签约；化工园区环氧丙烷、双氧水等丙烯产业链项目竣工投产。加大产业布局调整，金珠工业区晨鸣纸业新厂建成使用；哈达湾服务业集中区列入国家老工业基地搬迁改造试点并加快启动，企业搬迁和对价谈判取得积极进展。推进项目进区，实施产业项目350项，万丰工业园、聚源40万吨聚醚、万科松花湖国际度假区、大仝软件外包服务基地等一批重大项目开工建设，一汽吉林G4、20万吨ABS通用料等120个项目投产运营，精细化工、汽车零部件、信息服务、休闲旅游等各具功能区特色的产业集群发展态势加速形成。

　　中心城市承载功能持续增强。完成城建投资276亿元，房地产和建筑业增加值分别增长12.8%和11%。实施重点城建工程207项，火车站东广场、解放中路立交桥、人民医院二期等123个项目竣工使用。长吉北线扩建工程顺利推进，绕城高速全线通车，新建改建各级各类公路496.5公里。棚户区改造及房地产开发1021万平方米、竣工500万平方米。维修改造路桥56项，铺设天然气管线57.2公里，生活污水集中处理率达到93.2%。实施城市管理标准化建设，建筑市场规范化管理及专项整治收到实效，交通畅通、城市绿化等工程实施受到群众好评，城市环境质量和整体形象得到进一步提升。

　　县域发展取得新成果。突出县域开发区建设，磐石“两区三园”、桦甸“五大园区”等产业集中度提升，维依康生物科技、山能集团油页岩综合开发等一批重点项目入区建设，资源型城市转型步伐加大，县域生产总值、地方级财政收入分别增长10%和19.4%。加快城镇化建设，县城基础设施不断完善，公共服务能力有效增强，新增建成区1.4平方公里；金珠、岔路河等省市试点镇基础设施、农民新居、产业支撑和外在形象发生新的变化，吉舒、天岗等一批特色城镇建设稳步推动。

　　（三）全力保障和改善民生，和谐稳定社会局面持续巩固

　　总投资126亿元的122项民生事项如期完成。城镇新增就业9.7万人。建设廉租房和公租房11万平方米，1.5万户困难群众享受到租赁补贴。完成“暖房子”工程改造864万平方米，8.8万户居民受益。新农合重大疾病报销范畴增加到40种，实行单病种定额结算50种，最高支付限额提高到10万元；城镇居民在一、二、三级医院住院报销比例各提高5个百分点。连续7年第9次上调低保标准，城市低保提高到每人每月370元，农村低保提高到每人每年2350元，补差水平处于省内前列。治理教育“三乱”收到实效。探索社会化居家养老服务模式，新增养老机构床位2200张。又解决近15万农村人口饮水安全问题。全力组织抗洪抢险救灾，恢复重建水毁水利设施701处，114户受灾居民入冬前迁入新居。

　　社会事业协调发展。义务教育“阳光分班”积极推行，城区中小学食堂大宗食品原材料实施统一采购，新建改造校舍102万平方米，高新北区职业教育园区启动，餐饮职教集团、江城化工职教集团挂牌成立。基本药物制度和基层运行机制进一步完善，市中心医院门诊综合楼投入使用，村卫生室年度建设任务按时完成，重点疾病防治网络在全省率先建成，医疗保障服务能力有效提升。全面完成省下达的人口控制指标。人民剧院和全民健身中心建设顺利，铁道职业技术学院、吉林书画城建成使用。松花江河灯文化节被评为国家公共文化服务体系示范项目。高水平举办中国龙舟公开赛等大型赛事活动，深入开展各类群众性文体活动，人民群众的精神文化生活更趋丰富多彩。

　　平安吉林建设进一步加强。连续组织开展五轮安全生产隐患大排查大整改行动，全面推行网格化管理和安全生产标准化建设，积极构建安全生产长效管理机制，安全监管实现制度化、规范化和常态化。完善维护群众权益工作机制，强化领导接访和包案制度落实，一批重点信访问题得到妥善解决。加强社会治安综合治理，严厉打击各类违法犯罪行为，人民群众社会安全感进一步增强。国防后备力量持续加强，双拥共建活动深入开展。

　　（四）加大改革创新步伐，制约发展瓶颈因素有效破解

　　实施简政放权。取消和调整行政审批项目99项，向城区、开发区下放城市建设领域审批和管理事权62项。推进金融创新。落实金融业奖励政策，激励银行业金融机构扩大信贷规模，全市本外币贷款余额增长18%；有效整合政府性资产，探索组建金融控股集团和国有资产运营公司，三大融资公司和十大功能区实现融资160亿元；中信银行吉林分行和环城农村商业银行实现运营，新增小贷公司和担保公司20家。促进科技成果转化。在全省率先搭建科技成果交易平台，定期组织科技成果交流转化推介活动，企业、科研单位和金融机构实现有效对接，实施市级以上重大科技成果转化和重点科技攻关项目134项，100种新产品实现规模化生产。推进体制机制创新。松花湖旅游区、中新食品区管委会完成组建，高新北区、岔路河综合配套改革启动实施，集体资产公司化改革、土地流转等人口城镇化政策措施稳步推动。加大“上争”力度，争取到国家循环经济示范城市、肉菜流通追溯体系等一系列示范试点，在节能减排、电子商务城市、低碳城市、城市矿产基地等先行先试方面取得积极进展。全年累计争取国家和省各类财政资金140亿元以上。

　　各位代表，过去一年取得的成绩来之不易，这是市委科学决策、正确领导的结果，是市人大、市政协大力支持、有效监督的结果，是全市上下共同努力、奋发拼搏的结果。在此，我代表市人民政府，向全市450万人民，向辛勤工作在各条战线的广大建设者，向各级人大代表、政协委员、各民主党派、工商联和社会各界人士，向中省直单位、驻吉部队指战员和武警官兵，向始终关心支持吉林市发展的海内外朋友，表示衷心的感谢和崇高的敬意！

　　我们也清醒地认识到，发展中还存在不少问题：受经济下行、去产能化和我市现有产业结构特征影响，一些骨干企业生产经营困难、效益下滑、贡献率降低，一些重点建设项目未能如期投产达产、投资质量和效益不高，保持经济持续较快发展的压力较大。尽管近些年结构调整取得很大进展，但以原材料生产为主的产业格局、民营经济不发达的状况还没有从根本上得到改变。财政结构性矛盾突出，增收难度加大，可用财力增长难以满足以改善民生为核心的刚性支出不断增加的需要。政府职能转变还不到位，审批环节多、收费项目多且弹性大，一些部门和干部服务意识不强、工作作风不实、推动发展能力不足等现象还不同程度地存在。对于这些问题，我们一定高度重视，认真负责地加以解决。

　　二、2014年工作安排

　　今年是实施“十二五”规划和“三年看吉林、实现新跨越”的关键一年。我们将深入贯彻党的十八届三中全会、省委十届三次全会和市委十二届八次全会精神，按照中央和全省经济工作会议要求，把改革创新贯穿于经济社会发展各个领域各个环节，坚持“稳增长、调结构、促改革、惠民生”，全力推动创新发展、统筹发展、绿色发展、开放发展和安全发展，努力让城乡居民生活得更加美好。

　　主要预期目标：地区生产总值可比增长10%，规模工业增加值增长12%，全社会固定资产投资增长15%，社会消费品零售总额增长13%，地方级财政收入增长18%，城镇居民人均可支配收入和农民人均纯收入增长12%；节能减排完成省下达任务。

　　（一）以提高经济运行质量和效益为核心，全力以赴“稳增长”

　　正确处理速度与质量的关系，全力推进经济稳中求进、稳中求质、稳中求效。

　　抓好项目建设和招商引资工作。有效优化投资结构。实施“产业升级百项重点工程”，服务业、战略性新兴产业投资比重分别提高到40%和10%以上；亿元以上重大项目力争达到500项，占全市投资总额的45%以上。推进建成项目投产达效。全面清理近五年来建成的重点项目，分类施策，逐项落实生产条件，实现投产一批、达产一批、转产一批，提高产出效益。全年实现达产率70%以上。加强项目管理。创新项目建设推进机制，注重提高投资强度和产出效果，把生态保护和节能减排作为强制性条件，严格执行项目环境影响评价和节能评估审查制度；围绕保障性住房、养老、节能环保、产业升级等重点领域，做好项目谋划、储备，全力争取国家和省的支持。提高招商引资质量。围绕产业结构调整，瞄准世界500强及知名企业，充分发挥各地商协会作用，强化点对点招商、以商招商、专业招商等方式，有针对性地开展各类招商活动，进一步提高招商项目的合同履约率、资金到位率和项目开工率。新开工3000万元以上招商项目350项，新落地项目到位资金260亿元。

　　突出发展民营经济。强化示范引领。重点抓好50户企业集团和50户骨干民营企业发展壮大，培育100户成长性强的中小企业和100户实缴税金超千万元的重点企业，着力打造一批名牌品牌产品，带动民营经济整体水平快速提升。强化集群发展。围绕基础化工和精细化工、汽车零部件、软件及云计算等9个产业集群，积极引进、培育和发展一批配套企业和项目，提高链式化、终端化发展水平。全年新增“三上”企业300户以上、产值超亿元企业50户以上。强化创业服务。大力推进全民创业，推出2000个创业项目，积极拓展创业平台；进一步完善中小企业社会化服务体系，从企业设立、成长、壮大给予多方面政策支持，全面营造促进民营经济发展的社会氛围。

　　扶助企业加快发展。加强涉企服务。以规模工业企业为重点，引导企业顺应需求变化，调整产品结构，提高产品性能和质量，扩大市场份额；开展降本增效活动，大力压缩“三项费用”；组建行业联盟，抱团发展，引导企业集群式开拓域外市场，提升现有企业市场竞争力和抗风险能力；对减亏和增盈潜力较大的重点企业，分市县两级落实包保责任，重点亏损企业力争再减亏10亿元以上。推动整合重组。发挥工业资源、资产优势，推进现有企业与国内外大集团、大公司合资合作；采取技改投入、出租出售等方式，盘活存量资产和闲置土地，激活停产、半停产企业，努力实现资产效益的最大化。强化科技创新。加快电力电子、精细化工等公共技术研发中心建设，推动大专院校、科研单位技术与企业资本的深入对接，提高科技成果本地转化率，引导企业成为科技创新投入的主体。新产品规模化生产100种以上。

　　切实加强财税增收工作。提高收入质量。全力培植地方性财源，强化重点税种征收管理，提高税收比重，促进地方可支配财力的持续增加。深化综合治税。完善综合治税信息平台建设，加强重点税源跟踪监控，推进个体税收社会化管理，加大依法治税力度，努力实现应收尽收。壮大总部经济。不断完善总部经济政策保障体系，加大对现有总部企业服务力度，努力扩大总部在外的中省直企业就地缴存份额，再引进一批总部经济企业进驻我市，逐步实现引进总部税收向引进总部实体经济的转变。合理控制债务规模。进一步规范举债融资行为和工作程序，科学确定融资资金使用方向，有效监控债务结构和分布，努力防控和化解政府债务风险。

　　（二）以统筹产业发展、区域发展、城乡发展为重点，坚定不移“调结构”

　　第一，积极调整产业结构。加快构建现代产业体系，三次产业结构努力调整到8.5：49.5：42。

　　做优做强工业。按照新型工业化要求，推进产业创新试点城市建设。继续改造提升传统产业。积极化解产能过剩矛盾，推进钢铁行业向精深加工、上下游延伸发展，促进水泥行业等量置换、更新改造，支持石化、汽车、冶金、建材等行业实施跨区域战略联合重组、协作配套；完成冀东水泥搬迁，加快中钢吉炭、中钢吉铁等企业搬迁改造进程；改进设备和生产工艺，开展能耗物耗对标达标试点，技改投资占工业投资的比重达到45%。壮大农产品加工业，实施大润德农业综合开发等20个重点项目，培育产值超亿元龙头企业45户，带动种植业和养殖业产业化发展。大力发展战略性新兴产业。推进新材料、先进装备制造、电力电子、新能源、生物技术等产业规模化生产，谋划发展通用航空、医药健康产业，积极抢占未来发展制高点。建设吉林碳谷年产1000吨碳纤维等100个投资3000万元以上战略性新兴产业重点项目。战略性新兴产业产值占规模工业比重提升到29%以上。推动信息化与工业化深度融合。突出重点行业，着力拓展信息技术在产品研发、生产、营销、配送和售后服务等关键环节的应用；开展省级两化深度融合试验区建设，创建10户两化融合示范企业，实施15个信息化应用示范项目。

　　发展提升服务业。着力发展生产性服务业。重点围绕信息、服务外包、研发设计、物流等新兴产业发展，推进国家信息消费试点城市和电子商务示范城市建设，开工东忠、中讯软件园，加快微软IT学院、城市一卡通等项目建设，统泰一网全城购物中心项目上线运营；大仝软件服务外包基地竣工投产；推动龙潭化工物流园等省市服务业集聚区建设。积极发展文化旅游业。深度挖掘自然、历史、山水等文化内涵，加快松花湖、朱雀山、北大壶等旅游设施开发建设进程，打造四季各具特色的文化旅游产品，推动雾凇冰雪节等节庆活动形成产业链；发展休闲农业、乡村旅游，推出一批国家级休闲旅游农业星级企业和魅力乡村；加大宣传推介力度，不断扩大对外影响力和特色品牌优势，旅游业收入增长25%以上。提升商贸流通业档次。升级改造东北亚农产品批发市场等5个大型综合批发市场，批发业占社会消费品零售总额比重提高到20%；促进欧亚综合体、中凯红星家居建材港等一批重点项目尽快运营，发展一批具有现代交易模式的加盟店和连锁店，不断丰富新兴商贸业态。

　　稳定提高农业。强化农业基础地位，以发展现代农业为抓手，全面增强农业综合生产能力和市场竞争力。提高粮食和特产业规模化水平。积极推广高光效、良种培育等先进适用技术，集中推进90个粮油高产示范片建设，粮食总产量保持110亿斤阶段水平；发挥长白山特色资源优势，抓好食用菌、蔬菜等一批特色产业基地建设。推进现代畜牧业发展。新建、改扩建200个标准化养殖小区，规模化养殖比重达到85%以上；完善软硬件建设，保持无规定疫病区良好状态。加强品牌建设。深入宣传“中国粳稻贡米之乡”名片，扩大“万昌”、“舒兰”、“大荒地”等大米品牌影响力；推动甜粘玉米跨区联动发展，整合包装林蛙、人参、黑牛等长白山特色品牌，提高农产品附加值和市场占有率。创新农业经营模式。发展壮大村集体经济，加大对种养大户、家庭农场、农民专业合作社等新型农业经营主体的扶持力度，带动农业产业转型升级和规模经营。

　　第二，科学优化空间结构。坚持把十大功能区和县域作为今后一个时期发展的主要载体和战略重点，充分发挥开发区引领示范作用，持续加大工作推进力度。

　　推动十大功能区领先发展。年内新开发面积10平方公里以上。统筹高新北区、经开区、金珠工业区产业合理分工、协调发展。实施高新大路、金珠大桥、中新大街等基础设施项目50项，建设一汽低成本乘用车、大地化工资源综合利用、建龙钢铁6.5万千瓦煤气混合发电、万科松花湖国际度假区、保利北大壶综合开发等重点产业项目210项，十大功能区产业项目投资占全市比重提升到30%以上，地区生产总值、地方级财政收入、规模工业增加值占全市的比重分别达到29%、24%和45%。进一步创新体制机制，支持先行先试，申报建设保税物流中心，推进中新食品区、化工园区、金珠工业区晋级升位。

　　加快县域经济发展。围绕县域经济战略性调整，改造升级矿产开发、冶金机械、农产品加工等传统产业，发展壮大生物医药、新材料、现代物流等新兴替代产业，推进资源型城市转型；以县域开发区为主要载体，充分发挥当地资源禀赋，抓好修正医药产业园等一批县域工业重点项目建设，大力发展精深加工，提高产业集中度，快速形成县域特色化、集群式产业发展新优势。五县（市）生产总值、地方级财政收入分别增长11%和18%。

　　第三，努力调整城镇结构。以新型城镇化建设为引领，统筹城乡关系，加快形成以城带乡、城乡一体的发展格局。

　　加强中心城市建设。快速推进丰满大坝重建、东西部快速路、长吉南北线改造、吉哈高速等十个方面基础设施重点工程。加快哈达湾等老城区改造，抓好榆树沟搬迁改造，促进房地产业健康发展。全面做好交通畅通、物业管理、建筑市场管理等十个方面城市管理重点工作。强化地下管网改造和管护。提升重点街路、景观路美化绿化水平，解决小街小巷、老旧小区亮化问题。完善物业管理制度，强化弃管小区管理，分类推进小区物业管理规范化、标准化。加强静态交通，建成7处立体停车库，推进智能交通管理系统建设，进一步优化道路交通秩序。科学调整公交线网布局，配建公交基础设施，新投放公交车300辆，新建公交候车亭400个，解决部分偏远区域公交乘车难问题。加大建筑市场整治力度，进一步提高城市管理机械化、专业化水平，严格管理执法，提升城市管理的效率和质量。

　　推进县城和小城镇建设。坚持规划引领，加大基础设施建设和管护力度，加强城市管理，完备教育、文化、卫生等社会事业功能，大力发展第三产业，增加就业岗位，加快人口集聚步伐，打造五县（市）各具特色的县城新形象。以试点镇建设为突破口，继续抓好金珠、北大壶、孤店子、岔路河4个省级和越北、小白山2个市级示范城镇建设，再筛选一批重点镇纳入市级试点范畴，突出抓好产业发展和公共服务设施建设，尽快完善土地、户籍、就业、社保等方面的配套政策；以大荒地、恒成号等示范村为样板，扎实推动新农村建设，推进以人为核心的新型城镇化。

　　（三）以处理好政府与市场、企业、社会关系为导向，攻坚克难“促改革”

　　推进经济体制改革。在所有制改革方面，研究制定促进民营经济健康发展的配套改革措施；以管资本为主加强国有资产监管，完善国有产权、股权流动机制；建立市属国有企业董事会、监事会向市政府报告工作制度；探索国有资本收益上缴工作；稳妥推进厂办大集体改革。在市场体系改革方面，改革工商登记制度，降低市场准入门槛

；引导民营资本参与城市基础设施建设；建立完善兼顾群众基本生

活需求和资源要素节约利用的阶梯价格制度，逐步推进水、电、气、热等资源性产品价格改革。在金融体系改革方面，积极发展多层次资本市场，推动“新三板”和股权投资基金发展，扩大直接融资规模；发挥金融控股集团综合性金融服务平台作用，引进、支持社会资本创办各类金融机构和配套服务机构；壮大地方法人金融机构，推动都邦保险公司做大做强，深化农村信用社改革，支持市中小企业担保公司提高增信能力；打击非法集资等金融违法行为，加强信用体系建设，优化金融生态环境。在财税体制改革方面，推进“营改增”、资源税从价计征等改革，全面推行部门预算改革，逐步将政府性基金预算、社会保险基金预算和国有资本经营预算纳入财政预算管理。在土地制度改革方面，有序推动农村土地流转，做好农村集体建设用地

、农村宅基地确权登记发证

工作，

推进城乡统一的建设用地市场建设，积极探索农村经营性建设用地

与国有土地同等入市、同权同价；依法清理

城市闲置土地，提高土地集约节约利用水平。

　　推进社会领域改革。整合新型农村社会养老保险和城镇居民社会养老保险制度，拓展保障资金渠道。建立公开规范的公积金制度，改进住房公积金提取、使用和监管机制。有序放开教育领域，引导多种形式的民办教育机构规范发展。深化医药卫生体制改革，鼓励社会资本进入医疗卫生领域。完善文化市场准入与退出机制，健全现代文化市场体系，促进基本文化服务标准化、均等化。将生态环境保护与节约利用资源纳入审计监督范畴，从严考核环境效益指标。抓好安全监管防控体系试点建设，深化安全生产管理体制改革。健全社区服务体系，提升社区治理综合效能。做好产学研创新、城管体制创新等试点经验的承接和拓展。

　　推进行政体制改革。深入实施“简政放权”。向市场放权：实施负面清单管理，负面清单以外的内外资项目均可准入，引导社会资本参与公益性、经营性项目建设与运营，最大限度为各类市场主体松绑。向社会放权：推进行业协会商会与行政机关脱钩，落实好四大类社会组织向民政部门直接登记制度，逐步实现政府有关职能向社会组织转移。向基层放权：再向县（市）区集中下放一批审批权限，推进管理服务职能重心下移。改善政府服务和管理。由重审批向重服务转变，由“严进宽管”向“宽进严管”转变，着力规范市场秩序，创造公平竞争环境；进一步优化审批流程，减少审批环节，清理规范行政事业性和服务性收费，降低收费标准，加快公共资源交易中心和网上办事大厅建设，打造便民高效的政务服务平台。抓好政府机构改革。科学整合机构职能，规范机构设置，完成机构改革各项任务；继续推进事业单位分类改革，试行政府购买公共服务工作。

　　（四）以提升群众生活幸福感为己任，尽心竭力“惠民生”

　　将新增财力的80%以上用于改善民生，真正做到体现民意、惠及民利、赢得民心。

　　认真办好民生重要事项

　　（1）努力办好人民满意教育。实施幼儿教育覆盖工程，扩大学前教育资源；加强德育工作，保障学生身心健康；实施义务教育阶段按学区就近免试入学，扩大高中统招生比例，全面实行“阳光入学”、“阳光分班”，让更多学生平等享受优质教育；进一步治理教育“三乱”问题。

　　（2）努力办好人民满意的医疗卫生事业。采取综合性措施，切实降低常用药品、高值耗材和大型设备检查价格；扩大医保定点药店数量，进一步方便百姓；提高远程医疗的覆盖面和利用率，加大对基层医疗卫生机构的对口帮扶，提升乡镇卫生院、社区卫生中心和村卫生室标准化建设水平，加强三甲医院管理，着力解决群众看病贵、看病难问题。

　　（3）努力保障群众的“菜篮子”。加强政策支持和技术指导，提高棚室冬季利用率，延长本地蔬菜供应时间，采取综合措施降低流通成本，加大平价直销便利店建设力度，启动全国第四批肉菜流通追溯体系项目建设，为市民提供优质、充足的蔬菜。

　　（4）积极推进新型养老体制和机制建设。加强养老服务设施和网络建设，扩大居家养老服务试点范围，完善服务机制，拓展服务项目；鼓励社会力量创办养老机构，支持社会养老机构进社区，开展多元化居家养老服务；发挥公办养老机构托底作用，努力做到老有所养。

　　（5）大力支持创业、促进就业。加强创业基地建设，开发一批具有地方特色的创业项目，落实扶持政策，积极开展定单、定向等多种形式职业技能培训，促进群众自主创业和就业。

　　（6）完善困难群众帮扶长效机制。深入落实低保困难家庭再保障政策，在基本生活、医疗、就学、住房等方面加大政策优惠和特殊扶持力度，帮助再保障困难家庭解决实际问题。

　　（7）加大城市危房、棚户区改造和保障房建设力度。完成城市D级危房改造建设任务，年内改造棚户区100万平方米，建设廉租房6万平方米、公租房5万平方米，对1.25万户住房困难家庭发放廉租住房租赁补贴，更好地满足中低收入群体和困难家庭的住房需求。

　　（8）实施“暖房子”工程。继续推进既有居住建筑节能改造，努力实现建成“暖房子”城市目标；建立“暖房子”工程管理长效机制，巩固节能改造成果；加快热源热网建设，增加供热能力500万平方米以上。

　　（9）切实抓好安全生产。强化生产安全、消防安全、交通安全和食品药品安全，深入推进企业标准化建设，全面实施网格化监管，落实长效机制和主体责任，严格监督检查和隐患整改，实现安全发展。

　　（10）精心维护社会稳定。加快建设覆盖城乡的公共视频监控网络，完善社会治安防控体系，严厉打击多发性侵财犯罪和严重暴力犯罪活动；深入排查化解矛盾纠纷，妥善解决人民群众合理诉求；完善应急管理机制，增强突发事件预警和应急处置能力。

　　加强生态环境建设。把生态环境建设作为最重要的民生工程，努力建设天蓝、地绿、水净的美好家园。启动实施大气污染治理工程。加强空气质量监测分析，提高排放准入门槛，加大燃煤机组脱硫脱硝改造力度，进一步取缔分散供热锅炉，推进经营性锅炉达标排放，逐步迁移平房燃煤用户，严格废弃物焚烧管理，渐次淘汰黄标机动车并严禁转入落籍，进一步优化空气质量。加强水环境治理。深入推进松花江水污染防治和市区小流域治理工作，加大地下水保护和监管力度，妥善解决好生活性污水排放问题，逐步推广利用再生水资源，松花江出境断面保持国家三类水质标准。依法开展清收还林。做好宣传发动、调查摸底、全面清收、停耕还林等工作，当年清收83万亩，还林55万亩。力争通过3年的努力，清收166万亩、还林100万亩以上，全市森林覆盖率达到57%。深入推进节能减排。充分利用节能减排财政政策综合示范城市、低碳城市试点、城市矿产示范基地、循环经济示范城市等政策优势，推进化工、冶金、电力等高耗能行业技术升级、节能改造，完善垃圾收运体系和餐厨废弃物处理体系建设，加强资源的有序利用、废弃物的综合利用；新建六水厂，改造五水厂，完成污水处理厂二期工程；大力培育发展节能环保产业，积极推广绿色建筑，推进住宅产业化，加强节能技术研发和生物质等新能源应用，实现经济效益和环境保护的协调统一。

　　统筹做好其他重点工作。完善公共文化服务体系，推进社区文化活动室和乡镇文化站建设，加快建设全民健身中心、人民剧院等一批文化重点项目，深入打造松花江文体活动风景线等文化品牌，争创全国公共文化服务体系示范区，提升历史文化名城对外影响力。认真落实《全面推进依法行政实施纲要》，提高政府立法质量，规范行政执法行为，深入开展“六五”普法。完成第三次全国经济普查。做好人口和计生、妇女儿童、民族宗教、外事侨务、残疾人、气象、地震等工作。提高国防动员和后备力量建设质量，开展好各类社会化拥军活动，推动军民融合发展，争取全国双拥模范城“八连冠”。

　　各位代表，今年全市改革发展任务十分繁重。我们将以开展党的群众路线教育实践活动为契机，按照为民务实清廉的总要求，坚决贯彻中央“八项规定”，自觉接受市人大、市政协监督，下力气破解“四风一顽症”，大力弘扬清廉、敬业、和谐、务实、创新、学习“六种风气”，全力打造服务型政府和法治政府。

　　坚决整治形式主义，坚持实事求是，进一步端正学风、改进文风会风，把心思用在干事业上，把功夫下到出实招、办实事、求实效上，进一步树立求真务实的政府形象。

　　坚决整治官僚主义，坚持民主集中制，改进调查研究，深入实际、深入基层、深入群众，全心全意为人民做事谋利，进一步树立执政为民的政府形象。

　　坚决整治享乐主义，坚持“两个务必”，克己奉公，勤政廉政，始终保持昂扬向上、奋发有为的精神状态，进一步树立清正廉洁的政府形象。

　　坚决整治奢靡之风，坚持勤俭办事，严控“三公”经费支出，强化监察监督，努力降低行政成本，进一步树立勤俭节约的政府形象。

　　坚决整治“不落实”问题，坚持把抓落实作为做好工作的根本保障，严肃抓落实的责任，完善抓落实的机制，强化行政执行力，确保各项工作真正落到实处，进一步树立干事成事的政府形象。

　　各位代表，我市改革发展正站在新的历史起点上，做好今年工作意义重大、任务艰巨。让我们在省委、省政府和市委的正确领导下，在市人大、市政协的大力支持下，更加紧密地团结起来，锐意进取，奋发作为，全面开创科学发展、富民强市的崭新局面！
